# Poly(β-hydroxyl amine)s: Valuable Building Blocks for Supramolecular Elastomers with Tunable Mechanical Performance and Superior Healing Capacity

**DOI:** 10.3390/polym14040699

**Published:** 2022-02-11

**Authors:** Linlin Wang, Jie Zhou, Lei Li, Shengyu Feng

**Affiliations:** 1 Key Laboratory of Special Functional Aggregated Materials, Ministry of Education, School of Chemistry and Chemical Engineering, Shandong University, Jinan 250100, China; wll0218@sdu.edu.cn (L.W.); wll8810@sdu.edu.cn (J.Z.); fsy@sdu.edu.cn (S.F.); 2 Shandong Key Laboratory of Advanced Silicone Materials and Technology, Shandong University, Jinan 250100, China; 3 Key Laboratory of Colloid and Interface Chemistry, Ministry of Education, School of Chemistry and Chemical Engineering, Shandong University, Jinan 250100, China; 4 Weihai New Era Chemical Co., Ltd., Weihai 264205, China

**Keywords:** dual-cross-linked elastomer, supramolecular elastomer, poly(β-hydroxyl amine), self-healing

## Abstract

Supramolecular elastomers integrated with high mechanical toughness and excellent self-healing ability offer attractive applications in various fields such as biomedical materials and wearable electronics. However, the multistep preparation process for creating functional polymer precursors and the expensive stock materials required are two factors that limit the widespread use of supramolecular elastomers. Herein, for the first time, poly(β-hydroxyl amine)s generated by amine-epoxy polymerization were used in the development of supramolecular polymer materials. Based on the novel silicon-containing poly(β-hydroxyl amine)s synthesized by the polymerization between 1,3-bis(3-glycidyloxypropyl)tetramethyldisiloxane and 3-amino-1,2-propanediol, dually cross-linked supramolecular elastomers with both hydrogen bonding and metal coordination were achieved, displaying adjustable mechanical properties with the tensile strength varying from 0.70 MPa to 2.52 MPa, respectively. Thanks to the dynamic nature of the supramolecular interactions, these elastomers exhibited favorable hot-pressing reprocessability and excellent self-healing performance, with the healing efficiency reaching up to 98% at 60 °C for 48 h. Potential applications for photoluminescent materials and flexible electronic devices were demonstrated. We believe that its simplicity of synthesis, adjustable mechanical properties, and robust self-healing capacities bode well for future applications of this new supramolecular elastomer.

## 1. Introduction

Supramolecular elastomers [1] are elastic networks cross-linked by non-covalent bonds such as hydrogen bonds [2,3], metal–ligand interactions [4,5], host–guest interactions [6,7], and π-π stacking [8,9,10]. Due to the reversible nature of non-covalent interactions, supramolecular elastomers generally have excellent thermoplasticity, enhanced processability, and self-healing properties. Supramolecular elastomers are usually prepared based on one specific supramolecular interaction. Though the mono-cross-linking design is quite straightforward, the tunability of the material properties is limited by the variety of supramolecular interactions. Depending on the strength of the intermolecular interactions, the mechanical strength of the obtained materials ranges from a few tenths of MPa to tens of MPa, while the healing occurs with or without external triggers. In order to improve the design flexibility, mechanical toughness, and self-healing properties of polymeric materials, a cooperative double cross-linking strategy has recently been explored by combining two orthogonal supramolecular interactions [11,12]. For example, both metal-coordinated bonds and hydrogen bonds have been introduced into the same polymer network, and the resulting elastomer can heal itself at room temperature while exhibiting excellent elasticity [13]. By combining strong and weak metal–ligand interactions through incorporating two potential ligands of Fe^3+^ ions, a dual-cross-linked polydimethylsiloxane network was reported, exhibiting tunable mechanical strength and self-healing ability [14]. In addition, dually associated supramolecular polymers containing both hydrogen bonding and metal coordination was demonstrated with enhanced fracture energy and exhibiting multiple shape-memory and self-healing properties [15]. Due to their rich chemical structures, adjustable mechanical properties, and self-healing capacities, supramolecular elastomers can potentially be used in the field of wearable electronics, electronic skins, motion tracking, and health monitoring [16,17,18]. Despite these great advances, the multistep preparation process for creating functional polymer precursors and the expensive stock materials required are two factors that limit the widespread use of supramolecular elastomers.

The polymerization between amine and epoxide functionalities is known to proceed under ambient conditions to provide poly(β-hydroxyl amine)s. This reaction is catalyst-free and generates no by-product [19]. Its functional group tolerance allows for the synthesizing of a library of polymers with various functional groups from many commercially available amines. Because of these advantages, amine–epoxy polymerizations have been applied to synthesize multifunctional main-chain cationic polymers [19], hyperbranched polymers [20], porous materials [21], shape-memory materials [22], and zwitterionic hydrogels [23]. Our group has utilized this reaction to develop novel clickable and fluorescent silicon-containing poly(β-hydroxyl amine)s, which have been employed in reusable adhesives and reprocessable elastomers [24]. Poly(β-hydroxyl amine)s possess abundant hydroxyl groups with intrinsic hydrogen-bonding capabilities. Furthermore, repetitive nitrogen atoms in the polymeric backbone can provide lone pairs of electrons for coordination with electron-deficient metal ions. Both features make poly(β-hydroxyl amine)s promising candidates for the preparation of supramolecular polymer materials. This information inspired us to explore the use of poly(β-hydroxyl amine)s to design dually supramolecular elastomers with unique dynamic properties, such as self-healing and reprocessable capacities.

In this study, we designed a new type of silicon-containing poly(β-hydroxyl amine)s via the amine-epoxy polymerization between 1,3-bis(3-glycidyloxypropyl)tetramethyldisiloxane and 3-amino-1,2-propanediol. The obtained polymer possesses the low glass transition temperature of −22 °C, as well as abundant hydroxyl side groups. Due to the cumulative effect of weak hydrogen-bonding interactions, a stretchable, rehealable, and reprocessable hydrogen-bonded silicone elastomer was achieved. Additionally, introducing copper chloride resulted in a dual-cross-linked network formed by hydrogen bonds (hydroxy groups) and coordination bonds (Cu^2+^ with nitrogen atoms) [25,26], endowing our material with enhanced and adjustable mechanical properties. Thanks to the dynamic features of both supramolecular interactions, an excellent self-healing capacity was achieved in these dual-cross-linked elastomers. This research provides a feasible method for designing mechanically tough, self-healing, reprocessable materials that demonstrate promising applications for use in photoluminescent materials and flexible electronics. To our knowledge, this is the first attempt to use poly(β-hydroxyl amine) generated via the amine-epoxy polymerization for supramolecular materials.

## 2. Materials and Methods

### 2.1. Materials

1,3-Bis(3-glycidyloxypropyl)tetramethyldisiloxane (97%) was purchased from Hwrk Chemical Co., Ltd. (Beijing, China). 3-Amino-1,2-propanediol (98%) was supplied by Heowns Biochemical Technology Co., Ltd. (Tianjin, China). Copper chloride (anhydrous) was obtained from Energy Chemical Co., Ltd. (Shanghai, China). Europium nitrate hexahydrate (99.99%) and chloroform-D (CDCl_3_, 99.8%) were purchased from Aladdin Co., Ltd. (Shanghai, China). Methanol (AR grade), tetrahydrofuran (THF, AR grade), petroleum ether (AR grade), and chloroform (AR grade) were provided by Fuyu Fine Chemical Co., Ltd. (Tianjin, China). Multi-walled carbon nanotubes with an outer diameter of 4–6 nm (MWCNT) were obtained from Organic Chemicals Co., Ltd. (Chengdu, China) All the reagents and solvents were used as received.

### 2.2. Characterization and Measurements

The proton nuclear magnetic resonance (^1^H NMR) spectrum was analyzed on a Bruker Avance 400 MHz spectrometer at 25 °C with CDCl_3_ as the solvent and without tetramethylsilane (TMS) as the internal reference for chemical shifts (δ, ppm). The Fourier transform infrared spectrum (FT-IR) was recorded on a Bruker TENSOR 27 infrared spectrophotometer using the KBr pellet technique from the 4000 cm^−1^ to 500 cm^−1^. The resolution was 4 cm^−1^ and 16 scans with a heating rate of 3 °C/min. Gel permeation chromatography (GPC) indices were detected with a Waters 1515 liquid chromatograph equipped with a 2414 refractive index detector. The column temperature was set at 40 °C and the flow rate of THF was stable at 1.0 mL/min. Differential scanning calorimetry (DSC) was measured using the TA Instruments SDTQ 600 under dry nitrogen flow, and the temperature range was from −140 °C to 100 °C, with a heating and cooling speed of 10 °C/min. For each specimen, two cooling-heating cycles were applied to eliminate thermal history, and the T_g_ was obtained from the second cooling-heating process. The fluorescence (excitation and emission) spectra were performed on a Hitachi F-4500 fluorescence spectrophotometer using a monochromatic Xe lamp as an excitation source. Ultraviolet absorption spectra in a CH_3_OH solution were detected using a TU-1901 Dual Beam UV-Vis spectrophotometer in the wavelength range of 200–700 nm. The transmittance spectra of the elastomer were also recorded by a TU-1901 Dual Beam UV-Vis spectrophotometer in the wavelength range of 200–700 nm. Narrow X-ray photoelectron spectroscopy (XPS) was performed using a Thermo Fisher Scientific (Waltham, MA, USA) ESCALAB 250 spectrometer with a monochromatic Al Kalph (150 W) source. The survey and high-resolution scans were generated at 200 eV pass energy and 30 eV pass energy, respectively. Scanning electronic microscopy images (SEM) and EDS (including elemental mapping) were obtained via a Quanta 250 FEG-SEM device (FEI, Lausanne, Switzerland), and the contents of some elements were recorded simultaneously. The specimens were cut and coated with a thin layer of platinum black before the investigation. Mechanical tensile tests (including the healing and reprocessing tests) of the elastic films were implemented using the Instron 3343 material testing system with a speed of 100 mm/min. For all the tensile tests, the films were cut into several dumbbell-shaped samples with a size of 40 × 4 × 0.8 mm^3^, and at least three parallel tests were conducted. The resistance of the conductive material during the stretching process (speed rate = 5 mm/min) was measured step by step using a CHINT ZTY0105A digital multimeter.

### 2.3. Synthesis of Polymer P1 via the Amine-Epoxy Polymerization

The mixture of 1,3-bis(3-glycidyloxypropyl)tetramethyldisiloxane (7 mmol, 2.544 mL) and 3-amino-1,2-propanediol (7 mmol, 0.638 g) was added to methanol (3 mL), and stirred with a mechanical stirrer at 60 °C for 24 h. Subsequently, the crude mixture was diluted with a small amount of THF and was slowly precipitated into a large amount of petroleum ether, repeated three times. After vacuum drying, the target **P1** was obtained. (*M*_w_ = 10,130, PDI = 2.10.)

### 2.4. Preparation of the Hydrogen-Bonded Elastomer

The solution of polymer **P1** in methanol was poured into a Teflon plate mold, dried naturally at room temperature, then dried in a vacuum at 80 °C for 24 h. Finally, the transparent hydrogen-bonded elastomer was obtained.

### 2.5. Preparation of Dually Supramolecular Elastomers (**P1-Cu-x/1**)

Various amounts of CuCl_2_ were introduced into the **P1** network to yield dually supramolecular elastomers named as **P1-Cu-x/1**, where the molar ratio of nitrogen atoms vs. CuCl_2_ in **P1** was x/1. The preparation of elastomer **P1-Cu-3/1** is described as an example. To the solution of polymer **P1** (2.26 g, 5 mmol of nitrogen atoms, 1 eq.) in 2 mL of methanol, the solution of CuCl_2_ (22.4 mg, 0.167 mmol, 0.33 eq.) in 1 mL of methanol was added. After stirring for 5 min, the mixture was poured into a Teflon mold, dried naturally at room temperature, then dried in a vacuum at 80 °C for 24 h to remove the solvent. Finally, a blue stretchable film was obtained. Following a similar process, all the other dually supramolecular samples were prepared by adjusting the molar ratio of CuCl_2_ vs. nitrogen atoms.

### 2.6. Hot-Pressing of the Supramolecular Elastomers

The fractured materials of the elastic films were collected together and introduced into a steel mold (150 × 100 × 0.5 mm^3^), which was pressed at 50 °C for 20 min under 4 MPa. The tensile measurement was then performed to evaluated mechanical property of the material.

### 2.7. Self-Healing Tests of the Supramolecular Elastomers

The elastomer **P1** or **P1-Cu-6/1** was cut into dumbbell-shaped specimens with a size of 40 × 4 × 0.8 mm^3^. Each specimen was cut into halves using a blade. The two cut-off pieces were then instantly rejoined at the fracture surfaces and kept at either room temperature or 60 °C for different durations. Tensile measurements on the healed samples were conducted to quantitatively evaluate the self-healing efficiency, which is defined as the ratio of the tensile strength of the healed and original samples.

### 2.8. Preparation of the Photoluminescent Supramolecular Elastomer **P1-Eu-x**

Various amounts of Eu(NO_3_)_3_·6H_2_O were introduced into the **P1** network to yield photoluminescent supramolecular elastomers named as **P1-Eu-x**, where the molar ratio of Eu(NO_3_)_3_·6H_2_O vs. nitrogen atoms in **P1** was x. Using the preparation of elastomer **P1-Eu-20%** as an example, to the solution of polymer **P1** (1 g, 2.21 mmol of nitrogen atoms, 1 eq.) in 12 mL of methanol, the solution of Eu(NO_3_)_3_·6H_2_O (0.22 g, 0.442 mmol, 0.2 eq.) in 2 mL of methanol was added. After stirring at 40 °C for 4 h, the mixture was poured into a Teflon mold, dried naturally at room temperature, then dried in a vacuum at 80 °C for 24 h to remove the solvent. Finally, a stretchable film was obtained. Following a similar process, all the other photoluminescent supramolecular elastomers were prepared by adjusting the molar ratio of Eu(NO_3_)_3_·6H_2_O vs. nitrogen atoms.

### 2.9. Preparation of the Conductive Supramolecular Elastomer P1-MWCNT

To the mixture of 3.0 g of hydrogen-bonded polymer **P1** in a certain amount of methanol, 10 wt% of multi-walled carbon nanotubes (MWCNT) was added uniformly and dispersed by ultrasound (50 °C, 1.5 h). Afterwards, the mixture was poured into a Teflon mold, dried naturally at room temperature, then dried in a vacuum at 80 °C for 24 h to remove the solvent. Finally, the conductive supramolecular elastomer **P1-MWCNT** was obtained. 

## 3. Results

**Scheme 1:**
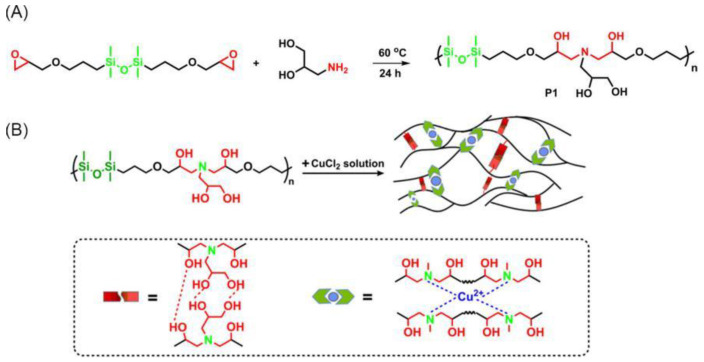
(**A**) The synthesis of silicon-containing poly(β-hydroxyl amine) **P1** via the amine-epoxy polymerization between 1,3-bis(3-glycidyloxypropyl)tetramethyldisiloxane and 3-amino-1,2-propanediol. (**B**) The preparation of supramolecular poly(β-hydroxyl amine) elastomers cross-linked with both hydrogen bonds (red polygons) and coordination bonds (green arrows and blue circles).

Based on our previous work, poly(β-hydroxyl amine) was selected as the parent polymer to construct dual-cross-linked supramolecular elastomers. As shown in Scheme 1A, this functional polymer is readily prepared via the click polymerization of amine and epoxy monomers at ambient conditions. Along with its polymeric backbone, hydroxyl groups can form hydrogen bonds with each other, while nitrogen atoms with lone-pair electrons can effectively coordinate with metal ions. Both types of non-covalent interactions identify poly(β-hydroxyl amine) as a perfect candidate for constructing supramolecular materials (Scheme 1B). The mechanical behaviors of the obtained networks can be rationally tuned by regulating the metal ion content. Meanwhile, the dynamic natures of the hydrogen bond and the coordination bond give the elastomer outstanding self-healing capabilities.

### 3.1. Synthesis and Characterization of Polymer **P1**

**Figure 1:**
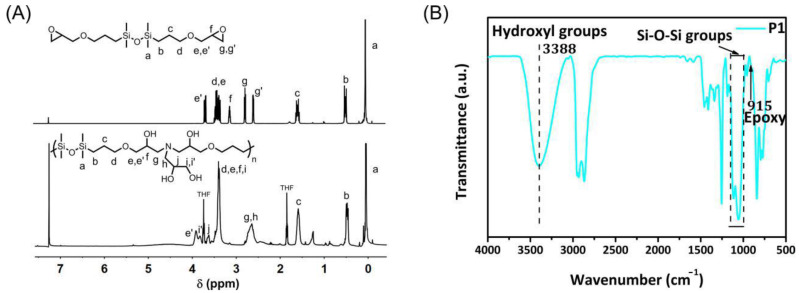
(**A**) The ^1^H NMR spectra of 1,3-bis(3-glycidyloxypropyl)tetramethyldisiloxane (top) and polymer **P1** (bottom). (**B**) The FT-IR spectrum of polymer **P1**.

The parent polymer was synthesized by the amine-epoxy polymerization between commercially available 1,3-bis(3-glycidyloxypropyl)tetramethyldisiloxane and 3-amino-1,2-propanediol. The former monomer with flexible siloxane units was selected to endow the obtained polymer with a low glass transition temperature, while the latter monomer with hydroxyl groups was selected to increase the density of the hydrogen bonds. The obtained polymer **P1** was thoroughly investigated using ^1^H NMR, FT-IR and GPC. Figure 1A shows the ^1^H NMR spectra of monomer A and polymer **P1**. In the spectrum of 1,3-bis(3-glycidyloxypropyl)tetramethyldisiloxane, the characteristic peaks of the epoxy protons appeared at 2.59, 2.78, and 3.13 ppm, which disappeared after polymerization with 3-amino-1,2-propanediol. In the spectrum of **P1**, the proton resonances from the methylene groups (g, h) adjacent to the nitrogen atoms overlapped at 2.44–2.82 ppm, while those from the methyl groups (a) adjacent to the silicon atoms appeared at 0.01–0.07 ppm, indicating that the two target motifs were successfully introduced into the polymer chains. The integration ratio of the typical proton signals (a:(g,h):(d,e,f,i):j:i′:e′ = 12:6:9:1:1:2) was in accordance with the theoretical calculation, further confirming the expected polymer structure. Moreover, the FT-IR measurements in Figure 1B showed that the two stretching absorption peaks of the primary amine groups (approximately 3500 and 3400 cm^−1^) in 3-amino-1,2-propanediol and the peak of the epoxy groups (915 cm^−1^) in 1,3-bis(3-glycidyloxypropyl)tetramethyldisiloxane disappeared after the polymerization. In addition, the GPC analysis of **P1** showed a unimodal distribution with an *M*_w_ of 10,130 g/mol and a polydispersity index of 2.1. These results confirm the successful synthesis of **P1** via amine-epoxy polymerization.

**Figure 2:**
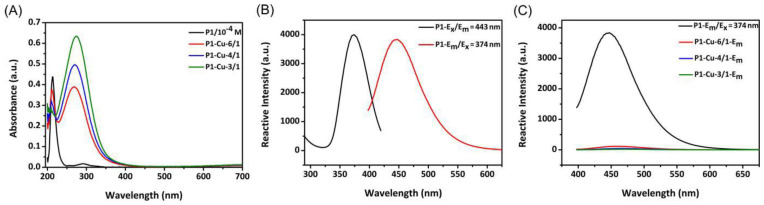
(**A**) The UV-vis spectra of polymers **P1**, **P1-Cu-6/1**, **P1-Cu-4/1**, and **P1-Cu-3/1** in CH_3_OH ([N atom], 10^−4^ M). (**B**) The fluorescence excitation and emission spectra of polymer **P1** in CH_3_OH ([N atom], 10^−4^ M). (**C**) The fluorescence emission spectra of polymers **P1**, **P1-Cu-6/1**, **P1-Cu-4/1**, and **P1-Cu-3/1** at the same excitation wavelength in CH_3_OH ([N atom], 10^−4^ M).

Due to the presence of nitrogen atoms on the backbone, polymer **P1** is expected to possess excellent coordination capabilities with electron-deficient metal ions. Therefore, UV-vis spectroscopy was used to investigate its coordinating behavior with CuCl_2_ (Figure 2A). The UV-vis spectrum of polymer **P1** exhibited a major absorption peak at around 231 nm and a minor one at around 291 nm. With the addition of CuCl_2_, the former peak gradually decreased, while the latter almost disappeared. More importantly, a new absorption peak appeared at around 270 nm, and its intensity constantly increased with the addition of CuCl_2_. The difference in UV-vis spectra indicates the change in the electronic environment of the nitrogen atoms and the new UV absorption band could be ascribed to the bonding σ(N) → antibonding d_x2–y2_(Cu) charge transfer (according to the literature), demonstrating the effective coordination between polymer **P1** and the Cu^2+^ ions [25,26]. 

Unexpectedly, polymer **P1** emits blue fluorescence under UV light at 365 nm, even though it does not possess a conventional chromophore. Therefore, the excitation and emission spectra of **P1** were studied to verify its fluorescent behavior. As illustrated in Figure 2B, the excitation wavelength appeared at 374 nm, and the emission wavelength appeared at 447 nm. This unconventional fluorescent phenomenon can be explained by the presence of N→Si coordination bonds in the polymeric backbones [27,28]. Photoluminescence would be generated by the through-space charge transfer from the lone electron pair in the nitrogen atoms to the 3d empty orbital of the silicon atoms. Moreover, the N→Cu^2+^ coordination interactions could lead to new charge transfer, thereby weakening the original N→Si coordination bonds, possibly inducing fluorescence quenching. To confirm this conjecture, fluorescent emission spectra were recorded after introducing Cu^2+^ into the **P1** solution in methanol. As shown in Figure 2C, the fluorescence emission intensity clearly decreased with the increase of the Cu^2+^ content, indicating that this fluorescence quenching behavior was closely related to the N→Cu^2+^ coordination property of polymer **P1**.

### 3.2. Hydrogen-Bonded and Metal-Coordinated Elastomers

Since there are hydrogen-bonding interactions between hydroxyl groups and between hydroxyl groups and nitrogen atoms in polymer **P1**, it is expected that these interactions might behave as physical crosslinking points to provide hydrogen-bonded elastomers. In addition, introducing strong metal coordination bonds into the weak hydrogen bonding network would lead to hierarchical non-covalent bond networks, allowing the flexible adjustment of elastomer properties. Upon deformation, weak hydrogen bonds tend to effectively dissipate energy by dynamic association and disassociation, while the strong coordination bonds can maintain high tensile strength. Therefore, a series of **P1** elastomers with various contents of CuCl_2_ were prepared.

**Figure 3:**
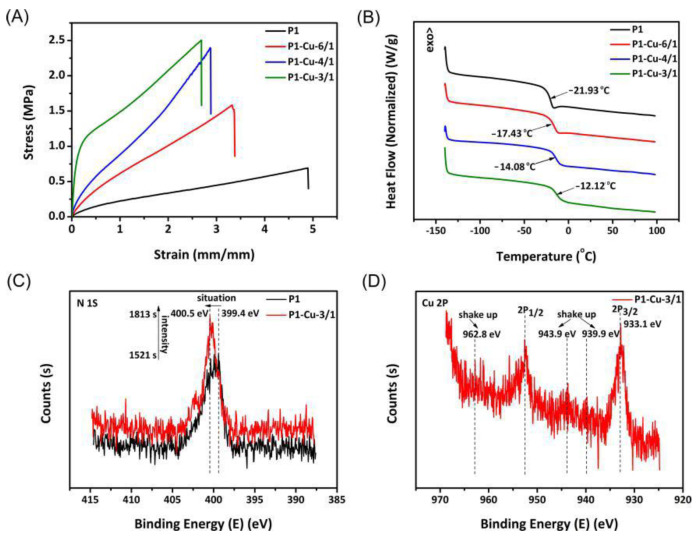
(**A**) The stress–strain curves and (**B**) differential scanning calorimetry curves of supramolecular elastomers **P1** and **P1-Cu**. (**C**) The X-ray photoelectron spectrum of elastomers **P1** and **P1-Cu-3/1** in the N 1S region. (**D**) The X-ray photoelectron spectrum of elastomer **P1-Cu-3/1** in the Cu 2P region.

According to the stress–strain curves shown in Figure 3A, the **P1** elastomer showed the tensile strength of 0.70 MPa and the elongation at break of 490%, demonstrating the effective hydrogen-bonded crosslinking. Then various amounts of CuCl_2_ were introduced into the **P1** network to yield dually supramolecular elastomers named as **P1-Cu-x/1**, where the molar ratio of CuCl_2_ vs. nitrogen atoms in **P1** was 1/x. Upon adding 1/6 eq. CuCl_2_ (vs. nitrogen atoms) to the polymer network, the tensile stress of the **P1-Cu-6/1** sample was greatly increased up to 1.59 MPa, with the decrease of elongation at break to 337%, indicating the formation of a more compact network with higher crosslinking density resulting from the metal coordination. The mechanical properties of these elastomers varied greatly with the CuCl_2_ molar ratios. When the amount of CuCl_2_ increased to 1/4 eq., the tensile stress of the elastomer **P1-Cu-4-1** reached 2.41 MPa, and the maximum tensile strain reached 287%. With an increase of CuCl_2_ to 1/3 eq., the large number of CuCl_2_ ions led to a heavily cross-linked polymer network. As a result, the **P1-Cu-3-1** sample showed a tensile stress as high as 2.52 MPa, but the tensile strain decreased significantly to 268%. Meanwhile, the introduction of CuCl_2_ also substantially increased the toughness and Young’s modulus (E) of the elastomers. Among them, the **P1-Cu-3-1** elastomer showed the highest toughness of 4.52 MJ/m^3^ and E of 4.69 MPa. These results demonstrate that the mechanical properties of these elastomers can be flexibly adjusted by tuning the N/CuCl_2_ molar ratio in the polymer network.

In addition to the mechanical properties, the thermal properties of the obtained elastomers were also significantly affected by the Cu/N coordination. Due to the adequate flexibility of the siloxane backbones, the hydrogen-bonded elastomer **P1** possesses a low glass transition temperature (T_g_) value; however, the introduction of Cu^2+^ can increase the degree of the crosslinking obtained elastomers, thus leading to the restriction of chain motion and hence, the increase of its T_g_. The DSC results in Figure 3B showed that the T_g_ of **P1** was found at −21.93 °C, whereas those of the metal-coordinated elastomers **P1-Cu-6/1**, **P1-Cu-4/1** and **P1-Cu-3/1** were measured at −17.43 °C, −14.08 °C, and −12.12 °C, respectively. In addition to hydrogen bonding, the presence of coordination in the **P1-Cu** samples was believed to decrease the mobility of the polymer chains, therefore increasing the T_g_ values. Meanwhile, no crystalline formation was observed for any elastomer under the studied experimental conditions, likely due to the poor crystallization ability of polymer **P1**.

To demonstrate the coordination between the nitrogen atoms and copper ions in the **P1-Cu** elastomers, narrow XPS analysis using a high resolution was applied to measure the change in binding energy after adding CuCl_2_ to the hydrogen-bonded elastomer. For **P1-Cu-3/1**, Figure 3C revealed that both the binding energy and intensity in the N 1S region were greater than those of **P1**. Furthermore, there was a shoulder peak at 934.0 eV for Cu 2p _3/2_ peaks (Figure 3D), which could be attributed to the Cu/N coordination complex, and the shake-up peaks at 939.9–943.9 eV and at 962.8 eV indicated the presence of partly coordinated Cu^2+^ [29,30]. The charging of the electron cloud environments of the N and Cu atoms indicated that the lone pair electrons of the nitrogen atoms transferred to the empty orbitals of Cu^2+^ ions, contributing to the physical crosslinking of the obtained elastomers.

**Figure 4:**
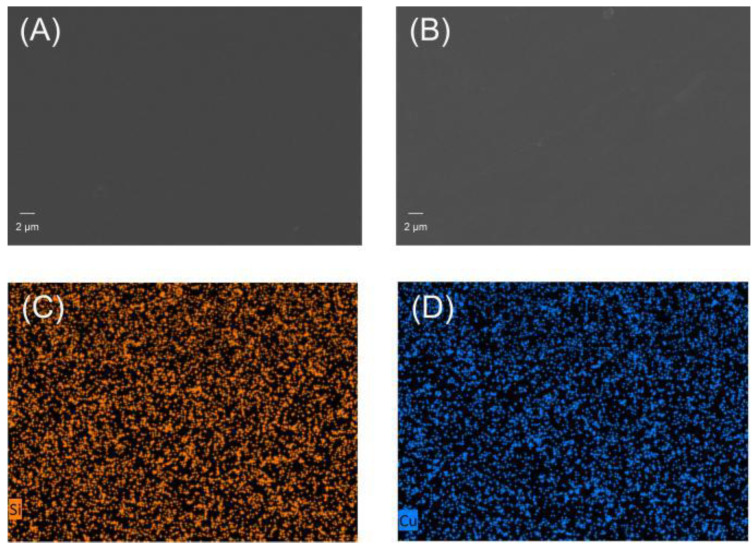
SEM images of elastomers **P1** (**A**) and **P1-Cu-3/1** (**B**). Elemental mappings of Si (**C**) and Cu (**D**) in elastomer **P1-Cu-3/1** via EDS.

The surface morphology holds important theoretical and practical significance for elastomers; therefore, the surface topographies of **P1** and **P1-Cu-3/1** were observed via SEM. Figure 4A,B showed that the introduction of coordination bonds did not affect the morphology of elastomers. A continuous and uniform surface morphology was observed, with no evidence of microphase separation, holes, or aggregation. This demonstrated that the coordination bonding between the poly(β-hydroxyl amine) matrix and metal ions was considerably strong, and high compatibility was observed inside the elastomers [31]. In addition, the element mapping obtained by EDS indicated that copper was successfully introduced into the supramolecular network and evenly distributed within the elastomer, similar to silicon (Figure 4C,D).

### 3.3. Dynamic Properties of the Supramolecular Elastomers

**Figure 5:**
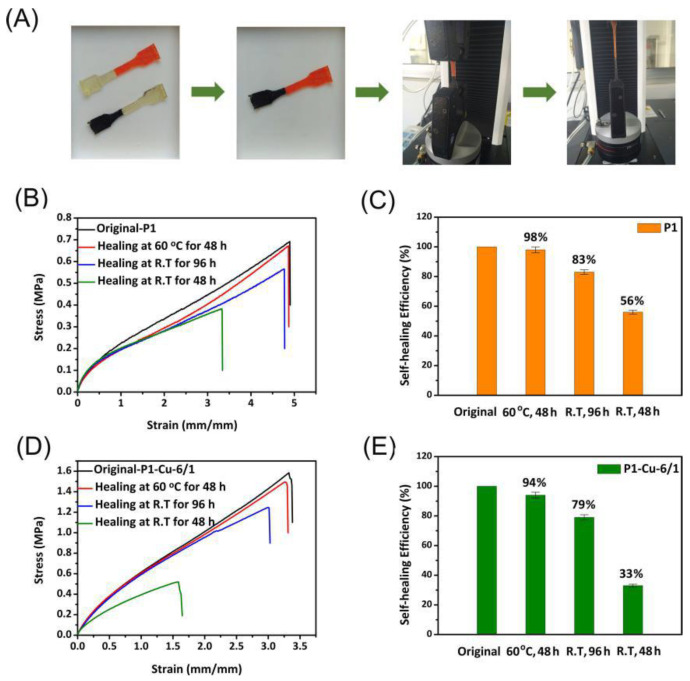
(**A**) Pictures of the self-healed elastomer **P1** and its tensile process. (**B**) The tensile stress–strain curves of the original and healed **P1**. (**C**) The self-healing efficiency of **P1**. (**D**) The tensile stress–strain curves of the original and healed **P1-Cu-6/1**. (**E**) The self-healing efficiency of **P1-Cu-6/1**. (RT—room temperature).

Considering the reversibility of the hydrogen bonds and coordination bonds, along with the excellent flexibility of polymer **P1**, both the hydrogen-bonded elastomer **P1** and the metal-coordinated **P1-Cu** are expected to possess self-healing capabilities. To prove this hypothesis, the **P1** or **P1-Cu-6/1** specimen was cut in half using a blade. The obtained cut-off pieces were then rejoined at the fracture surfaces and incubated at either room temperature (RT) or 60 °C for different durations. Figure 5A clearly showed that the ruptured surface was completely healed, withstanding the tensile stress. The typical tensile stress–strain curves of the pristine and healed **P1** and **P1-Cu-6/1** elastomers are shown in Figure 5B,D, respectively. Their self-healing efficiency is defined as the ratio of the tensile strength of the healed sample to that of the pristine sample. 

After being held at RT for 48 h, the tensile strength and the strain at break of elastomer **P1** were 0.38 MPa and 340%, respectively. As expected, the healing efficiency increased with the incubation time, manifested by 83% of tensile strength recovered after 96 h (Figure 5C). Heating was more effective in increasing the self-healing efficiency, which reached up to 98% at 60 °C for 48 h. Nearly complete recovery of the original tensile curve was observed in Figure 3B, demonstrating the excellent self-healing capability of **P1**. In contrast, the healing efficiency of **P1-Cu-6/1** decreased to 94% under the same conditions (Figure 5E) because the higher crosslinking degree led to a greater restriction of segmental movement. 

**Figure 6:**
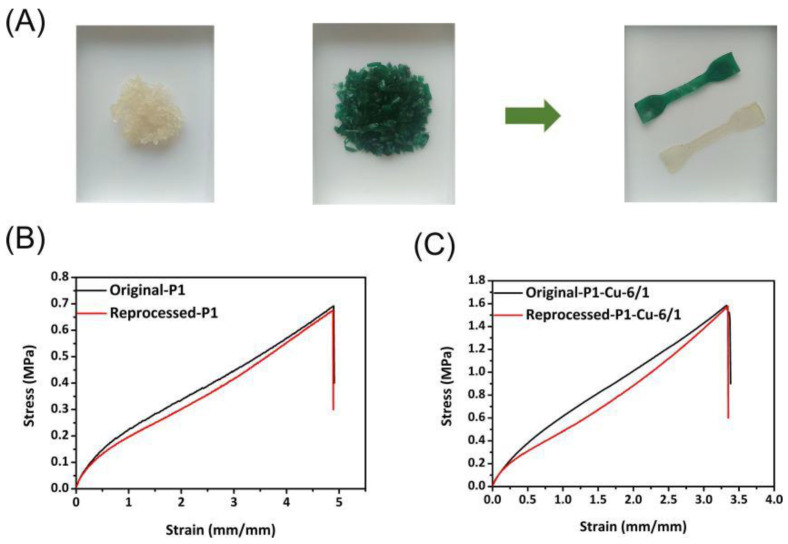
(**A**) Pictures of samples **P1** (white) and **P1-Cu-6/1** (blue) before and after hot-pressing at 50 °C for 20 min under 4 MPa. (**B**) The tensile stress–strain curves of the original and reprocessed **P1**. (**C**) The tensile stress–strain curves of the original and reprocessed **P1-Cu-6/1**.

In addition to self-healing abilities, both **P1** and **P1-Cu** elastomers also exhibit excellent reprocessability, benefiting from the reversibility of the supramolecular interactions. For the reprocessing experiment, we placed the **P1** elastomer pieces in a steel mold and then pressed it at 50 °C under 4 MPa for 20 min. After cooling to room temperature, the obtained film was cut into dumbbell-shaped samples and subjected to tensile tests to evaluate its mechanical properties (Figure 6A). As shown in Figure 6B, the tensile properties of **reprocessed-P1** were found to be nearly the same as those of the original **P1**, illustrating its excellent reprocessability. Meanwhile, although the metal coordination reduced the mobility, or flexibility, of the main chains, the networks containing metal ions still displayed favorable reprocessing properties. For example, after hot-pressing, the *σ*_max_ and *ε*_b_ of **P1-Cu-6/1** were found to be 1.58 MPa and 334%, respectively (Figure 6C), which were nearly identical to the original values (1.59 MPa, 337%).

### 3.4. Applications for Photoluminescent and Conductive Supramolecular Elastomers

Given the integration of robust mechanical strength, superior extensibility, and excellent self-healing capability, both types of supramolecular elastomers show promising potential for use in photoluminescent materials and flexible electronic devices, such as strain sensors.

Lanthanide ions have a long-lived excited state and strong narrow-width emission bands in the visible region resulting from their f–f transitions [31,32]. Therefore, they are widely used in the preparation of photoluminescent materials such as fluorescent tubes, lasers, field-emission displays, and optical amplifiers. In this study, supramolecular elastomers **P1-Eu** with a photoluminescence capacity were successfully prepared based on the coordination of Eu^3+^ ions with the polymer **P1**. Different amounts of Eu(NO_3_)_3_·6H_2_O were introduced into the **P1** network to obtain photoluminescent supramolecular elastomers named **P1-Eu-x**, where the molar ratio of Eu(NO_3_)_3_·6H_2_O vs. nitrogen atoms in **P1** was x.

**Figure 7:**
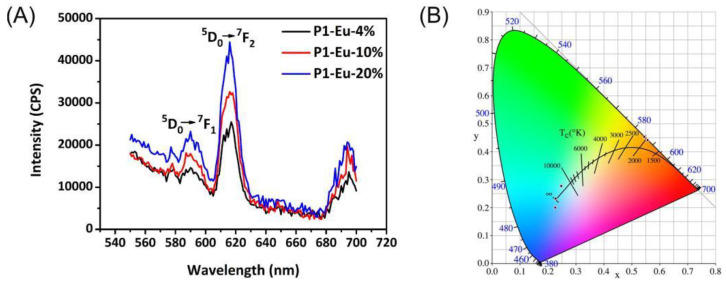
(**A**) The fluorescence emission spectra of elastomers **P1-Eu-4%**, **P1-Eu-10%**, and **P1-Eu-20%** at the same excitation wavelength (365 nm). (**B**) The CIE1931 RGB colorimetric diagram of samples **P1-Eu**. The small black dots represent the three kinds of **P1-Eu** samples with different N/Eu^3+^ ratios.

Their solid-state fluorescent spectra were measured at the excitation wavelength of 365 nm. As shown in Figure 7A, the three elastomers **P1-Eu-4%**, **P1-Eu-10%**, and **P1-Eu-20%** all exhibited the characteristic emission peaks of Eu^3+^ ions at 591 nm and 617 nm. The former peak was assigned to the 5D_0_→7F_1_ transition of the Eu^3+^ ions, while the latter resulted from the 5D_0_→7F_2_ transition. The emission intensities at 591 and 617 nm varied with the N/Eu^3+^ ratios. In addition, the very strong emission peak at 617 nm illustrated the highly polarized chemical environment of the Eu^3+^ ions. As demonstrated above, the **P1** elastomer itself would also exhibit the emission at 443 nm under the excitation of 365 nm. Therefore, the photoluminescent colors of the **P1-Eu** samples resulted from both the Eu^3+^ ions and the **P1** elastomers. As shown in Figure 7B, the emission color change could be easily observed in the CIE-1931 RGB coordinates, which were calculated from the emission spectra. As the Eu^3+^content increased, these materials showed a continuous color change from blue to cyan, and finally, to bright green.

**Figure 8:**
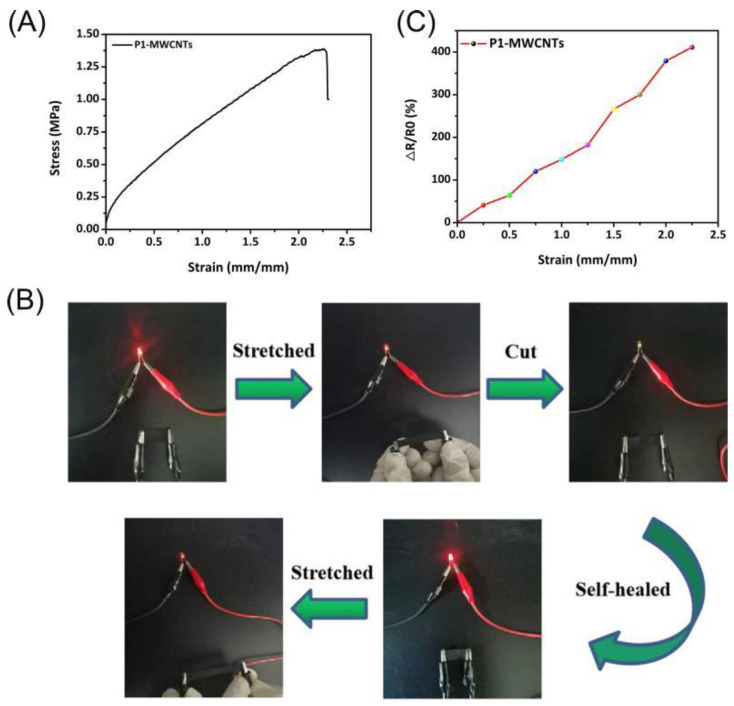
(**A**) The tensile stress–strain curve of elastomer **P1-MWCNT**. (**B**) The LED illumination (7 V) via the **P1-MWCNT**-based electrical circuit; The LED luminance change during the stretching (ε_b_ ≈ 50%) and damaging processes of the **P1-MWCNT**; The LED illumination and the response to stretching based on the self-healed elastomer. (**C**) The change in the electrical resistance of **P1-MWCNT** under different strains: ΔR/R_0_ value of the **P1-MWCNT** in response to tensile deformation (ΔR and R_0_ are the resistance variation upon stretching and the initial resistance, respectively).

Another potential application of elastomer **P1**, that is, for use in the production of self-healable electronic devices, was further investigated. A self-healing conductive elastomer, **P1-MWCNT**, was successfully prepared via the combination of hydrogen-bonded elastomer **P1** and multiwalled carbon nanotubes (MWCNT) (Figure 8A; σ_max_ = 1.39 MPa, ε_b_ = 231%). To qualitatively evaluate the electrical conductivity of the **P1-MWCNT** elastomer, a closed circuit consisting of a piece of **P1-MWCNT** (20 × 8 × 0.7 mm^3^), a small LED bulb, a regulated power supply (7 V), and some connecting wires was designed. As shown in Figure 8B, the LED lamp was instantly illuminated by switching on the power supply. When the sample was subjected to a tensile strain of approximately 50%, the luminous intensity of the LED lamp dropped correspondingly. When the elastomer was cut in half using a blade, the LED light immediately turned off. However, after the two cut surfaces made contact and self-healed, the LED lamp was again illuminated. Similarly, as the repaired elastomer continued to be stretched, the brightness of the LED lamp decreased due to the increase in resistance.

Next, to preliminary explore the response behavior of the **P1-MWCNT** to external strain, a piece of **P1-MWCNT** with an initial size of 20 × 8 × 0.4 mm^3^ was subjected to various strains to determine the change in resistance as ΔR/R_0_; ΔR and R_0_ refer to the resistance variation upon stretching and the initial resistance, respectively. Figure 8C reveals that the measured resistance value gradually increased with the increase in deformation. When the **P1-MWCNT** was stretched 225%, its ΔR/R_0_ value reached 410%. It was believed that the tight MWCNT would gradually become loose during the stretching process, which would destroy the conductive path and increased the resistance. According to the above results, it is reasonable to conclude that this supramolecular elastomer shows promising potential for future use in flexible strain sensors.

## 4. Conclusions

In summary, we have demonstrated that silicon-containing poly(β-hydroxyl amine)s synthesized via the polymerization between 1,3-bis(3-glycidyloxypropyl)tetramethyldisiloxane and 3-amino-1,2-propanediol are value building blocks for preparing stretchable, self-healable, and reprocessable supramolecular elastomers. Among the obtained elastomers, the hydrogen-bonded elastomer exhibits an unconventional fluorescence behavior owing to the unique Si–N coordination and displays a low T_g_ value resulting from the sufficient flexibility of the siloxane main chains. The mechanical properties of the metal-coordinated elastomers can be flexibly adjusted by varying the molar ratio of N/CuCl_2_. Moreover, these elastomers show excellent self-healing properties and hot-pressing reprocessability. Notably, we also prepared photoluminescence-tunable materials and self-healing conductive elastomers by combining the elastomers with Eu^3+^ and MWCNT, respectively. Considering the facile accessibility, adjusted mechanical properties, and outstanding dynamic capacities, these novel supramolecular elastomers based on poly(β-hydroxyl amine)s could find application for use as sustainable materials, fluorescent materials, and flexible electronic sensors.

## Data Availability

Not applicable.
